# The Effect of Crackers Enriched with Camelina Sativa Oil on Omega-3 Serum Fatty Acid Composition in Older Adults: A Randomized Placebo-Controlled Pilot Trial

**DOI:** 10.1007/s12603-023-1925-x

**Published:** 2023-06-03

**Authors:** R. De Giuseppe, I. Di Napoli, C. E. Tomasinelli, Alessandra Vincenti, G. Biino, E. Sommella, L. Ferron, P. Campiglia, F. Ferrara, P. M. Casali, H. Cena

**Affiliations:** 1 Laboratory of Dietetics and Clinical Nutrition, Department of Public Health, Experimental and Forensic Medicine, University of Pavia, via Bassi 21, 27100 Pavia, Italy; 2 Institute of Molecular Genetics, National Research Council of Italy, Pavia, Italy; 3 Department of Pharmacy, University of Salerno, Fisciano, Salerno, Italy; 4 FlaNat Research Italia Srl, Rho, Italy; 5 Laboratory Medicine Department - Centro Diagnostico Italiano, Milan, Italy; 6 Department of Public Health, Experimental and Forensic Medicine, University of Pavia, Pavia, Italy; 7 Clinical nutrition Unit, General Medicine, ICS MAUGERI IRCCS, Pavia, Italy

**Keywords:** Older adults, ageing, omega-3 fatty acids, Camelina sativa, alpha-linolenic acid

## Abstract

**Background:**

Camelina sativa oil is one of the richest dietary sources of omega-3, with polyunsaturated fatty acids amounts of over 50%, linolenic acid content of around 40–45%, and linoleic acid of about 15%. Moreover, this oil is a valuable source of antioxidants which provide oxidative stability. All those features raise interest in considering Camelina oil as an alternative and sustainable oil source providing stable omega-3-rich emulsions for functional food production.

**Objectives:**

The present study aimed to investigate the effects of Camelina oil-enriched crackers on serum omega-3 concentration, inflammatory markers and serum lipid profile.

**Design:**

Randomized placebo-controlled pilot trial. Setting: Research and Development Center (Complife Italia s.r.l.).

**Participants:**

Sixty-six free-living older volunteers (aged≥65 years).

**Intervention:**

Older adults were enrolled and randomly assigned to one of two groups: the camelina group or the placebo group. Subjects consumed daily 35 g of crackers (Camelina enriched crackers or placebo ones) twice daily for 12 weeks.

**Measurements:**

Serum polyunsaturated fatty acid profile, inflammatory status and serum lipid panel parameters were recorded pre and post-intervention.

**Results:**

In the camelina group, alpha-linolenic acid serum concentration was significantly higher (p<0.01) compared to the placebo group at the end of the study. Concerning inflammatory plasma markers, a significant mean pro-inflammatory interleukin-18 plasma concentration decrease in the placebo group compared to the camelina one was observed (p<0.05). No significant differences in other mean inflammatory markers concentrations post-intervention were noted in either group. Lastly, examining the change in lipid profile, it is noteworthy that a higher reduction of total cholesterol, low-density lipoprotein and triglycerides in the camelina group postintervention, despite the lack of statistical significance.

**Conclusion:**

Camelina oil significantly elevated the serum alpha-linolenic acid concentration with no significant changes in inflammatory markers and lipid profile.

**Electronic Supplementary Material:**

Supplementary material is available for this article at 10.1007/s12603-023-1925-x and is accessible for authorized users.

## Introduction

**O**ld age is characterized by a chronic and low-grade inflammatory state, named “inflamm-ageing” (1), which leads to metabolic dysfunction, physical limitations, frailty, and health impairment (2). Inflammageing is highly related to mild cognitive impairment and neurodegenerative disease (e.g., Alzheimer’s and Parkinson’s disease), atherosclerosis, heart disease, age-related macular degeneration, insulin resistance and type 2 diabetes, osteoporosis, sarcopenia and cancer (3, 4).

So far, literature has provided mixed results concerning the potential health benefits of long-chain omega-3 polyunsaturated fatty acids (ω-3 PUFA) namely eicosapentaenoic acid (EPA, 20:5 ω-3) and docosahexaenoic acid (DHA, 22:6 ω-3), as well as their precursor α-linolenic acid (ALA, 18:3 ω-3), in older population (5, 6) Indeed, despite the recognized capacity of ω-3 in reducing inflammation and promoting its resolution (4), studies evaluating ω-3 administration reported no effect compared to placebo (6, 7).

At the same time, the role of omega-6 polyunsaturated fatty acids (ω-6 PUFA), specifically linoleic acid (LA, 18:2 ω-6) and arachidonic acid (ARA, 20:4 ω-6), is still controversial (8, 9). Ω-3 and ω-6 are described as essential fatty acids because they cannot be synthesized by humans and must be provided by the diet. However, a small amount of EPA and, to a lesser extent, DHA can be derived from ALA, and ARA from LA (8, 10).

EPA and DHA are found mainly in oily fish (e.g., whiting, carp, mackerel, tuna, salmon, pollock, capelin and sardine) (11), as well as in meat and dairy products, albeit in a lower amount (12). However, recommendations for EPA and DHA dietary intake are not always satisfied because of unhealthy dietary choices, selective eating such as animal food avoidance, contamination with environmental pollutants overconcern, price and perceived unpleasantness of oily fish and fish oil (10).

Therefore, alternative dietary sources of ω-3, including vegetable oils, are needed (11). Foods high in ALAs include some seeds and seed oils (e.g. soybean, rapeseed, walnut and flax oil) and, among vegetable sources, camelina oil.

Camelina sativa, also known as camelina or false flax, is an ancient cultivated crop and underused Brassicaceae oilseed (13). Camelina oil is one of the richest dietary sources of ω-3, with PUFA amounts over 50%, ALA content around 40–45%, linoleic acid (LA, 18:2 ω-6) of about 15% and a valuable source of antioxidants, specifically tocopherols (13).

All those features raise interest in considering Camelina sativa oil as an alternative sustainable, affordable and scalable source of EPA and DHA well-suited to dietary and environmental needs as well as a promising alternative oil source providing stable omega-3-rich emulsions for food fortification and functional food production (14).

To test this potential alternative application of Camelina sativa oil on ω-3 serum concentration (i.g. ALA, EPA, DHA), a randomized placebo-controlled feasibility trial, was carried out on a group of free-living older adult volunteers (aged ≥ 65 years). The volunteers enrolled in the trial were asked to consume crackers prepared with Camelina sativa oil and therefore enriched with ALA, to test the efficacy of this functional food developed during the FoodNET (Food social sensor Network) project (15).

## Materials and Methods

### Outcomes

The present double-blind, randomized and placebo-controlled pilot study is part of the largest Food social sensor Network (FoodNET) project, funded by REGIONE LOMBARDIA - Research and Innovation, co-funded by POR FESR 2014–2020 (CUP E47F17000020009), and previously described elsewhere (15).

The study aimed at investigating in free-living older adult volunteers (aged ≥ 65 years) the effect of a functional food (e.g. crackers) prepared with Camelina sativa oil and consumed as a snack on:

i) serum polyunsaturated fatty acid profile (by measuring ALA: α-linolenic acid; EPA: eicosapentaenoic acid; DHA: docosahexaenoic acid; LA: linoleic acid; ARA: arachidonic acid levels) (primary outcome);
ii) inflammatory status parameters, paying particular attention to pro-inflammatory cytokines (by measuring IL-18: interleukin - 18; TNF-alpha: tumour necrosis factor-alpha levels); anti-inflammatory cytokines (by measuring TGF- beta 1: transforming growth factor-beta 1; TGF-beta 2: transforming growth factor-beta 2 levels) and C-Reactive Protein (CRP) levels (secondary outcome);
iii) serum lipid panel parameters (by measuring total cholesterol, TC; high-density lipoproteins cholesterol, HDL; low-density lipoproteins cholesterol, LDL; triglycerides, TG; concentrations) (secondary outcome).

### Participants

This randomized, placebo-controlled, double-blind pilot study was conducted according to the Declaration of Helsinki for Research on Human Subjects guidelines and approved by the Human Ethics Committee of the University of Milano-Bicocca (Milano, Italy). The study was also registered on ClincalTrials.gov, NCT04965948.

The study was conducted at Complife Italia s.r.l. (Milano, Italy) from June 2019 to March 2021.

One-hundred and thirty-five Caucasian subjects were considered eligible for the study, according to the following inclusion criteria i) aged ≥65 years; ii) free-living; iii) not on long-term regular vitamin/EPA or DHA supplementation; iv) not affected by hepatic and renal diseases, bleeding disorders; mental and neurological disorders; severe eating disorders; and exclusion criteria were i) known or suspected hypersensitivity to one or more ingredients of the snack; ii) institutionalized; iii) presence of certain diseases, including hepatic and renal disease, bleeding disorders; mental and neurological disorders, severe eating disorders; iv) subjects on long-term regular vitamin/EPA or DHA supplementation.

Out of 135 eligible subjects, 66 volunteers (21 male, 45 female) aged 64–80 years (70,6 ± 4,5 years of age) gave their consent to participate in the pilot study. At last, due to the inability to attend the follow-up, and Covid-19 restrictions, only 50 participants completed the protocol.

### Study design

**Figure 1:**
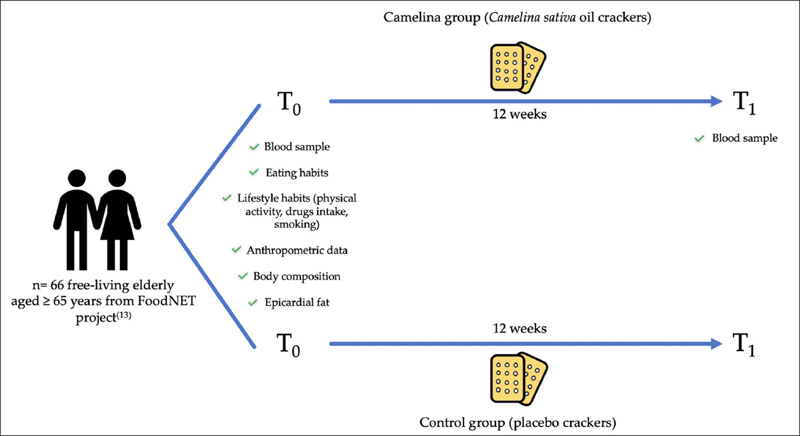
Study design T0, baseline; T1, after 12 weeks.

The participants were randomly assigned (1:1 ratio) into an experimental group or a control group, as shown in Figure. 1:

i) Camelina group (11M/23 F; mean age±SD: 71,08 ± 4,75 years);
ii) Placebo group (10M/22 F; mean age±SD: 70,03 ± 4,25 years).

Subjects in the Camelina group were instructed to consume Camelina sativa oil-enriched crackers (35 g twice daily for 12 weeks), while subjects in the control group were instructed to consume the same amount of placebo (same quantity of cracker without Camelina sativa oil for the same time) (Figure 1), without any dietary and lifestyle changes.

Personal identity and all personal medical information were kept confidential. Each participant was assigned a unique ID number. Participants were randomized using random numbers generated by a uniform distribution. Intervention and sample codification was hidden from both investigators and volunteers (double-blind study) and kept exclusively by the Principal Investigator (PI).

The energy and nutrient compositions of placebo and Camelina crackers are presented in Table 1.

**Table 1:** Average nutritional values for 100 g of Camelina sativa oil crackers and 100 g of placebo crackers

**Amount per 100 g**	**Camelina sativa oil crackers**	**Placebo crackers**
Energy	419 kcal (1770 kJ)	409 kcal (1723 kJ)
Fats	12 g	9.0 g
including saturated fatty acids	1.2 g	1.2 g
Carbohydrates	64 g	67 g
including sugars	0.9 g	2.4 g
Fibre	6.0 g	6.4 g
Proteins	10 g	12 g
Salt	2.2 g	2.5 g
	Ingredients: stone ground “type 2” soft wheat flour, Moradyn® corn flour (30%), camelina oil* (10%), Italian whole grain sea salt.	Ingredients: stone ground “type 1” soft wheat flour, wheat malt, corn oil, Italian whole grain sea salt.

*Average nutritional values for 100 g of Camelina sativa oil: Energy 460 kcal; Saturated fatty acids 10.09 g; Monounsaturated fatty acids 30.7 g; Polyunsaturated fatty acids 56.92 g; Linoleic acid 16.3 g; α-Linolenic acid 36.46 g; Erucic acid 3.34 g; Vitamin E 0.115 g.

In brief, pure cold-pressed Camelina sativa oil (nutritional values are presented in Supplementary Table S1) was obtained from seeds (Camelina Sativa L. Crantz REF. 18-C43-S) cultivated in Cremona (Italy) by FLANAT Research Italia (Rho - Milano, Italy). Crackers with Camelina sativa oil were formulated and manufactured by FLANAT Research Italia s.r.l. and packaged in transparent plastic bags containing 35 grams of crackers. A daily serving of Camelina sativa oil crackers provided approximately 2.6 g of ALA in toto. The placebo crackers formulation was chosen to match the carbohydrates, proteins, lipids, fibres, salt and energy (kcal) of Camelina sativa oil crackers. Crackers were packaged indistinguishably and labelled with the participant’s ID number.

Compliance with the study protocol was determined by assessing the uneaten cracker packages after 12 weeks. To prevent dropouts, a member of the research team was assigned the task to email, calling, and text messaging the participants during the whole study.

Anthropometric data (e.g., weight, height, waist circumference), body composition, epicardial fat, as well as dietary habits and lifestyle factors (e.g., physical activity, drug intake, smoking habit) were evaluated by professionally trained staff at baseline (T0) to describe anthropometry and lifestyle of the study population. Biochemical parameters, including serum polyunsaturated fatty acid profile, inflammatory status parameters and serum lipid panel parameters were measured at T0 and after 12 weeks of the functional food consumption (T1), to complete the assessment of nutritional status.

### Anamnestic and anthropometric data and dietary/lifestyle habits

All the volunteers were investigated both for chronic morbidities (i.e., type 2 diabetes, hypertension, hypercholesterolemia, osteoporosis, arthritis and chronic obstructive pulmonary disease) and 1ions.

Anthropometric parameters, including height (cm), weight (kg) and waist circumference (cm) were measured by standardized procedures as described elsewhere (16). Body mass index (BMI) was then calculated as the ratio of weight (kg) to height (squared meters) (16).

Body composition and phase angle were assessed with a standardized procedure using single-frequency (50 kHz) bioelectrical impedance analysis (BIA-101 model; Akern, Florence, Italy), with an alternating electric current at low intensity (800 µA). The calculated phase angle was considered an index of nutritional and functional status (17).

Eating habits were investigated using a validated self-administered dietary questionnaire(18). The questionnaire was originally drawn from the one developed and validated on an Italian youth population (18) and subsequently adapted by two dieticians to the adult population before its administration. The newly adapted version was previously tested on a sample of 24 subjects and revised accordingly (19).

The questionnaire assessed the daily consumption of typical foods and beverages of the local diet, such as bread, pasta, cereal products, fruits and vegetables, milk and yoghurt, tea and coffee, as well as the weekly consumption of meat and meat products, fish, eggs, cheese, legumes and sweets (18) and alcohol intake (18).

The questionnaire is multiple choice with a score ranging from 0 to 3 for each response, with the maximum score assigned to the healthiest one and the minimum score to the least healthy one according to the National Dietary Guidelines (20).

Physical activity of the previous 7 days was investigated by using the validated International Physical Activity Questionnaire, short-form (IPAQ; short form) (21), Italian version (22), and accordingly the metabolic equivalent of task (MET-min) per week was calculated as METs = MET level × minutes of activity × events per week. Moreover physical activity level was classified as sedentary (total METs < 699 kcal/kg/hour), moderate (total METs range 700–2519 kcal/kg/hour) and high (total METs > 2520 kcal/kg/hour) (21).

Smoking information was also collected. Volunteers were classified according to their smoking status at T0, as follows: smokers (current smokers), former smokers (if they had smoked in the past), and never smokers.

### Echocardiographic assessment of epicardial fat thickness

An echocardiographic assessment of epicardial adipose tissue thickness (EF) was conducted according to Iacobellis et al. (23) to provide additional information on visceral adipose tissue and cardiovascular risk (24). Volunteers were placed in the left lateral decubitus position. EF thickness was assessed on the free wall of the right ventricle in two-dimensional long and short heart axis views, at the end of the systole, using Vivid iq GE Healthcare (Milan, Italy) with a 3.5–7.5 MHz variable-frequency transducer.

### Sample collection and biochemical parameters evaluation

#### Sample collection

Blood samples were drawn at T0 and T1 in the morning, after an overnight fast. For the analysis of the biochemical parameters, blood specimens from each subject were collected in tubes, either with no additive (for serum) or containing EDTA to prevent coagulation (for plasma); EDTA specimens were centrifuged to obtain plasma samples.

Serum and plasma samples were divided into 4 aliquots (2 serum and 2 plasma aliquots) and stored at −20°C until the analysis of the inflammatory status (e.g. pro- and anti-inflammatory cytokines including IL-18, TNF-alpha, TGF- beta 1, TGF-beta 2, PCR; and C Reactive Protein, PCR) and the determination of polyunsaturated fatty acid profile (e.g. ALA, LA, EPA, DHA, ARA), respectively. The portion of serum that was not aliquoted and stored was immediately processed to assess lipid panel parameters (e.g. total cholesterol, TC; high-density lipoproteins cholesterol, HDL; low-density lipoproteins cholesterol, LDL and triglycerides levels, TG).

#### Serum lipid profile measure

Serum lipid parameter levels were measured at the Centro Diagnostico Italiano (CDI) S.p.A (via Simone Saint Bon 20, 20147, Milano, Italy) by routine method on Atellica ® CH Analyzer (Siemens Healthcare Diagnostics Inc., 511 Benedict Avenue, Tarrytown, NY, USA) with the relevant commercial reagent kits. According to the manufacturer, the linearity through the measurement range is between 10 – 550 mg/dL (0.11 – 6.22 mmol/L) for TG; 20 – 129 mg/dL (0.52 – 3.34 mmol/L) for HDL; 5.0 – 1000.0 mg/dL (0.13 – 25.90 mmol/L) for LDL; and 25–618 mg/dL (0.65 – 16.01 mmol/L) for TC.

The limit of blank (LoB), the limit of detection (LoD) and the limit of quantitation (LoQ) are 3 mg/dL (0.03 mmol/L), 10 mg/dL (0.11 mmol/L) and 4 mg/dL (0.05 mmol/L) for TG, respectively; 0.5 mg/dL (0.01 mmol/L), 2.7 mg/dL (0.07 mmol/L) and 2.1 mg/dL (0.05 mmol/L) for HDL, respectively; 1 mg/dL (0.03 mmol/L), 25 mg/dL (0.65 mmol/L), and 2 mg/dL (0.04 mmol/L) for TC, respectively; and an LoB of 0.8 mg/dL (0.02 mmol/L) and an LoQ of 3.0 mg/dL (0.08 mmol/L) for LDL.

#### Plasma polyunsaturated fatty acid profile

Plasma samples were thawed on ice, to 50 µL of the sample, 2.5 µL of an internal standard mixture (each standard concentration was 10 µg/mL in MeOH/H2O (80:20 v/v)) and 500 µL of ice-cold Hexane/2-Propanol (3:2) were added. Samples were vortexed at -20 °C for 10 mins and finally centrifuged at 14,680 rpm, for 10 mins at 4°C. 500 µL of supernatant were dried under nitrogen and were reconstituted in 1 mL of MeOH/H2O (80:20 v/v) and injected in UHPLC-MS/MS. ALA, α-linoleic acid-d14 (ALA-d14), LA, linoleic acid-d4 (LA-d4), EPA, Eicosapentaenoic acid-d5 (EPA-d5), DHA, Docosahexaenoic acid-d5 (DHA-d5), ARA, Arachidonic acid-d8 (ARA-d8) standards were purchased from Cayman Chemical Company (Michigan, USA). Other reagents were all purchased from Merck.

UHPLC-MS/MS analysis was carried out with a Shimadzu Nexera (Shimadzu, Milan, Italy) UHPLC system consisting of two LC 30 AD pumps, a SIL 30AC autosampler, a CTO 20AC column oven, a CBM 20A controller, and the system was coupled online to a triple quadrupole LCMS 8050 (Shimadzu, Kyoto, Japan) equipped with an Electrospray Ionization (ESI) source.

The separation was carried on a Kinetex® EVOTM C18 (100 × 2.1 mm; 2.6 µm, 100Å) protected with a SecurityGuardTM precolumn (5 × 2.1 mm) (Phenomenex, Bologna, Italy). The column temperature was set at 50°C, a flow rate of 0.5 mL/min was used, mobile phase consisted of (A): 5 mM CH3COONH4 in H2O (v/v) and (B): ACN. The following gradient was used: 0 min, 60%B, 6.5 min, 60%B, 6.6 min, 98%B, hold for 1 min, returning to 60% in 0.1 min. 2 were injected.

The ESI was operated in negative mode. Interface temperature, desolvation line temperature, and heat block temperature were set to 300 °C, 250 °C and 400 °C, respectively. Nebulizing gas, drying (N2) and heating gas (air) was set to 3, 10 and 10 L/min, respectively. MS/MS analysis was conducted in scheduled multiple reaction monitoring (MRM), and the transitions were optimized by flow injection analysis of the corresponding standard compounds.

Three replicates of each sample were performed. Samples were analysed in a random order to prevent any variation in mass spectrometer sensitivity and QC were randomly inserted in the batch to monitor system stability over time.

Calibration standards were prepared in MeOH/H2O (80:20 v/v) and internal standards were added to all calibration standards at concentrations of 25 ng/mL. Calibration curves in the range of 5–300 ng/mL for EPA, DHA and ARA, 5–500 ng/mL for ALA and 0.5–1.5 µg/mL for LA, with five concentration levels, were generated from regression analysis (R2 ≥ 0.999) and apply to quantitation of free fatty acids in plasma.

For sensitivity evaluation, limits of detection (LODs) and quantification (LOQs) were calculated by the ratio between the standard deviation (SD) and analytical curve slope multiplied by 3 and 10, respectively.

Repeatability was established by triplicate injections of QC samples with the same chromatographic conditions and analysed on the same day and within two consecutive days. Results were expressed as relative standard deviation % (RSD %) for concentration and retention time. Recovery was assessed by spiking a known amount of each internal standard solution at low, medium, and high concentration range in samples, which were subsequently extracted and analysed as previously described. The efficiency was calculated by comparing the internal standard peak areas of standards and real samples.

#### Serum inflammatory markers

Serum inflammatory status parameters (e.g. IL-18, TNF-alpha, TGF- beta 1, TGF-beta 2, PCR) were measured at Labospace s.r.l. (via Virgilio Ranzato, 12, 20128, Milano, Italy) by a bead-based multiplex assay (Luminex) on Bio-Plex® 3D Reader (Luminex FLEXMAP 3D, Bio-Rad Laboratories, Inc, Hercules, CA, United States) with the relevant commercial reagent kits.

The unknown sample cytokine concentrations were then calculated by Bio-Plex Manager software v6.0. using a standard curve derived from the known reference cytokine concentrations supplied by the manufacturer.

### Statistical Analysis

Summary statistics such as frequency, mean, standard deviation, median and range were used to describe collected data by the intervention group at baseline and follow-up. Continuous variables were checked for normality in their distribution. For each outcome variable, the difference between T1 and T0 values was computed and a t-test was used to assess variation in the mean. To take into account the effect of potential confounders, regression analyses were run setting outcomes as response variables, intervention group as a predictor and, smoking, eating habits or statin intake as confounders. Statistical significance was set at P<0.05.

The sample size of 26 subjects per group was calculated using standard formulas when the outcome measure is a continuous variable. A difference in ALA of 0.8 µg/mL was taken as the reference size effect (23), setting the probability of committing a type I error at a two-sided α < 0.05 and the power β > 0.80.

All statistical analyses were performed by STATA 16.1 (College Station, TX, USA).

## Results

### Study population and lifestyle habits evaluation

**Figure 2:**
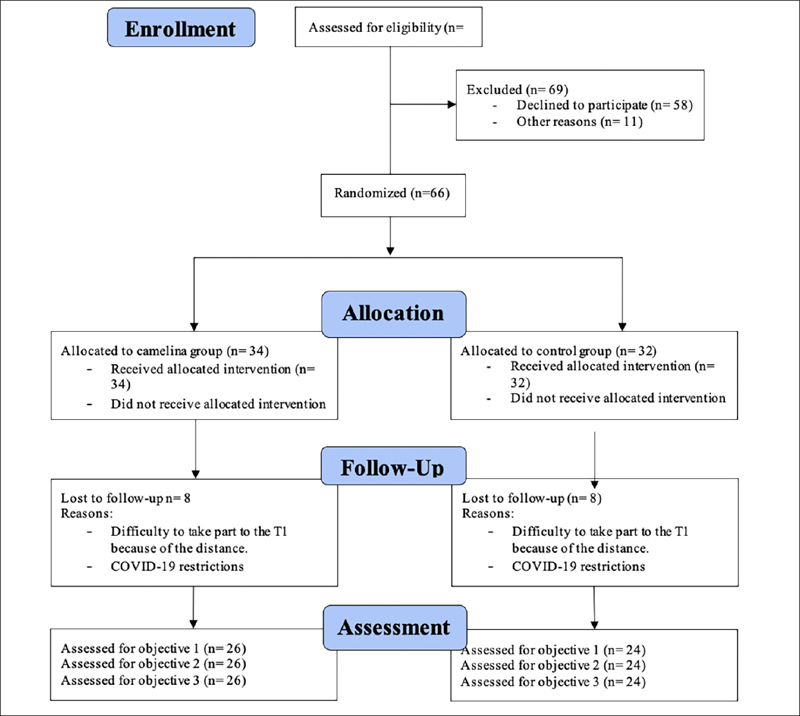
CONSORT (Consolidated Standards of Reporting Trials) flowchart for the study

Out of the 135 eligible free-living older adults aged ≥ 65 volunteers, only 66 (21 M/45 F; mean age±SD: 70,6±4,5 years) accepted to participate in the trial and were randomized as follows: 34 to the Camelina group and 32 to the placebo group. A total of 50 subjects completed the study, 14 dropped out because of Covid-19 containment measures, but they did not differ as to the collected data from those remaining in the study, therefore a per protocol analysis was adopted. The CONSORT (Consolidated Standards of Reporting Trials) flowchart for the study is presented in Figure 2.

Table 2 reported the general characteristics of the study population at T0 as a whole and by study group. The Camelina and placebo groups were well-matched for age, BMI, WC, EF, health and nutritional status (phase angle) (p>0,05). Overall, the mean BMI of the study population corresponded to overweight (26,6±4,04), while the mean phase angle (5,47±0,62) was within the range (5° to 7°) referred to healthy subjects.

**Table 2:** General characteristics and description of lifestyle parameters of participants included in the study (n = 66) at T0

		**Total (21M/45F)**	**Placebo group (10M/22F)**		**Camelina group (11M/23F)**		
	**N**	**mean (SD)**	**N**	**mean (SD)**	**N**	**mean (SD)**	**p-value**
Age (years)	66	70,6 (4,5)	32	70,03 (4,25)	34	71,08 (4,75)	0,346
BMI (Kg/m^2^)	66	26,6 (4,04)	32	26.2 (4.06)	34	27 (4.05)	0,423
WC (cm)	21	M 97,4 (9,1)	10	96,6 (8,2)	11	98,1 (10,2)	0,697
	45	F 90,1 (11,0)	22	89,8 (10,2)	23	90,4 (11,9)	0,842
Phase angle	56	5,47 (0,62)	28	5.5 (0,65)	28	5.4 (0,61)	0,399
EF	21	M 7,8 (2,5)	10	M 7,6 (2,3)	11	M 8,1 (2,7)	0,665
	44	F 8,33 (2,0)	22	F 8,2 (1,9)	22	F 8,3 (2,2)	0,940
Physical activity level (METs)	58	2345,69 (1613,75)	30	2284,3 (1399,45)	28	2411,4 (1839,98)	0,767
Eating habits (score)*	56	57,71 (6,88)	30	59,5 (6,77)	26	55,6 (6,51)	0,032
Health state (%)							0,780
1 chronic morbidity	22	33.3	10	31.2	12	35.3	
≥2 chronic morbidities	18	27.3	8	25.0	10	29.4	
None chronic morbidities	26	39.4	14	43.7	12	35.3	
Smoking habits (%)	58						0,534
Never smokers	33	56,9	18	60.0	15	53.6	
Former smokers	21	36,2	11	36.7	10	35.7	
Smokers	4	6,9	1	3.3	3	10.7	

BMI, Body mass index; WC, waist circumference; EF, epicardial fat; MET, metabolic equivalent of task; M, male; F, female; SD, standard deviation; N, number; %, percentage; Data were reported as mean ± SD, number (N) and percentage (%). T-test comparing mean values and Chi-square test comparing frequencies in placebo and Camelina groups.

Concerning dietary and lifestyle habits, no significant differences in physical activity levels and smoking habits were observed at T0. On the contrary, the placebo group showed significantly healthier eating habits than the Camelina one (59.5±6.77 vs. 55.6±6.51; p=0,032) (Table 2).

### Circulating fatty acids

Changes in plasmatic concentrations of ALA, LA, EPA, DHA and ARA at T0 and T1 in the placebo and Camelina groups are presented in Supplementary Table S2.

After 12 weeks of intervention, there was a significantly higher mean serum ALA concentration difference from T1 to T0 in the Camelina group compared to the placebo one (p <0.01) (Table 3). No significant difference was detected in mean LA, EPA, DHA and ARA concentration between groups before and post-intervention (p> 0.05) (Table 3).

**Table 3:** Mean difference of fatty acids at T1-T0

	**Placebo group**		**Camelina group**		
	**N**	**mean (SD)**	**N**	**mean (SD)**	**p-value**
ALA*	23	5.9 (4.75)	23	12.6 (9.32)	0.0046
EPA	23	0.9 (1.47)	25	2.5 (4.17)	0.0834
DHA	24	–1.1 (1.74)	26	–0.5 (1.66)	0.2307
LA	23	29.7 (35.83)	25	37.2 (42.86)	0.5189
ARA	23	5.4 (3.97)	25	4.7 (4.46)	0.5596

ALA, α-linolenic acid; EPA, eicosapentaenoic acid; DHA, docosahexaenoic acid; LA, linoleic acid; ARA, arachidonic acid; SD, standard deviation. Data were reported as mean ± SD, t-test comparing placebo and Camelina groups.

### Inflammatory response markers

Changes in serum IL-18, TNF-alpha, TGF- beta 1, TGF-beta 2 and CRP concentrations from T0 to T1 in the placebo and Camelina groups are presented in Supplementary Table S3.

After the intervention, the authors observed a significant decrease in mean IL-18 concentration in the placebo group compared to the Camelina group (p <0.05) (Table 4), with no significant differences in mean TNF-alpha, TGF- beta 1, TGF-beta 2 and CRP concentrations from T1 to T0 (p > 0,05) (Table 4).

**Table 4:** Mean difference of inflammatory response markers at T1-T0

		**Placebo group**		**Camelina group**	
	**N**	**mean (SD)**	**N**	**mean (SD)**	**p-value**
IL18*	24	–35.4 (51.6)	26	–5.4 (35.46)	0.0198
TNFα	23	7.6 (22.47)	24	13.3 (15.16)	0.3103
TGFβ1	23	–2497.1 (11474.48)	26	–5071 (10673.71)	0.4201
TGFβ2	23	–83.4 (174.22)	23	–69.4 (130.22)	0.7586
CRP	24	–505799.2 (735335.2)	24	–188277.9 (2030081)	0.4749

IL18, interleukin - 18; TNFA, tumor necrosis factor-alpha; TGFB1, transforming growth factor-beta 1; TGFB 2, transforming growth factor-beta 2; CRP, C-reactive protein; SD, standard deviation. Data were reported as mean ± SD, t-test comparing placebo and intervention groups.

Adjusted analyses showed no difference, even after considering smoking and eating habits as confounding factors.

### Serum lipid profile

Changes in serum concentrations of total cholesterol, its fraction, and triglycerides at T0 and T1 in the placebo and Camelina groups are presented in Supplementary Table S4.

As shown in Table 5, there were no significant differences in concentrations’ mean difference of TC, HDL, LDL and TG between T1 and T0 in the groups (p > 0.05) (Table 5).

**Table 5:** Mean difference of lipid profile at T1-T0

	**Placebo group**		**Camelina group**		
	**N**	**mean (SD)**	**N**	**mean (SD)**	**p-value**
TC	24	−0.5 (18.5)	26	−3.9 (27.22)	0.6085
HDL	24	−0.7 (5.22)	26	0 (4.75)	0.6578
LDL	24	0.7 (20.97)	26	−5.8 (26.08)	0.3436
TG	24	−4.9 (22.06)	26	−6.8 (23.77)	0.7724

TC, total cholesterol; HDL, high-density lipoproteins cholesterol; LDL, low-density lipoproteins cholesterol; TG, triglycerides; SD, standard deviation. Data were reported as mean ± SD, t-test comparing placebo and intervention groups.

Only 4 subjects (1 in placebo and 3 in camelina group) assumed statins. Adjusted analyses showed no difference in results, even after considering statins as a confounding factor.

## Discussion

The present randomized double-blind placebo-control study aimed to assess the impact of a functional food enriched with Camelina sativa oil on serum ω-3 concentration, inflammatory markers and serum lipid profile. A significant increase in ALA serum concentration was observed in the camelina group (p <0.01), while no significant difference was detected in EPA, DHA, LA and ARA serum concentrations post-intervention (p > 0.05).

Regarding ALA serum concentration in the experimental group, findings confirmed previous studies (25, 26). Karvonen H.M. et al. (26) reported an ALA plasma concentration increase after 30 g of cold-pressed camelina oil intake per 30 days (11.4 g/day of ALA) in 68 hypercholesterolemic adults (p < 0.001). Moreover, Schwab U.S. et al. (25) noticed that a 12-week diet enriched in 30 g camelina oil (10g/day ALA) improved serum ALA concentration compared to a fatty and lean fish diet (p <0.0001).

Although it is well-known that in the human organism, ALA is converted into EPA and DHA (27, 28), the postintervention slight increase in serum EPA concentration found in the experimental group of this study did not reach statistical significance (p = 0.0834).

As reported in the literature (27, 28), ω-3 and ω-6 fatty acids are synthesized from ALA and LA through biosynthetic pathways that share the same elongate and desaturase enzymes. Consequently, excessive consumption of LA or ALA in the diet may compete between and shift the balance towards the production of ARA or EPA and DHA, respectively (27, 28). By considering the eating habits of the whole sample, our results showed that the placebo group had significantly healthier eating habits than the experimental one (p < 0.05); therefore, it is suggestive that worse eating habits might correspond to ALA conversion into downstream metabolites.

On the other hand a significant mean pro-inflammatory IL-18 plasma concentration decrease in the placebo group, compared to the experimental one, was observed (p <0.05). Moreover, no significant differences in mean TNF-alpha, TGF-beta 1, TGF-beta 2 and CRP concentrations post-intervention (p > 0.05) were noted in either group.

Omega-3 PUFAs are generally known for promoting the resolution of inflammation(8), however, data from clinical trials on the role of ALA intake are conflicting. Indeed, Su H et al. (29) enquired about the effect of increasing dietary ALA intake on serum inflammatory markers and did not find any beneficial effect in healthy subjects. On the contrary, Haghighatdoost et al. (30), analyzing the effect of oral or intravenous ALA supplementation on serum inflammatory markers concentration, suggested a possible decreasing effect of ALA on inflammatory mediators.

Therefore, the lack of the expected effect of ALA supplementation on inflammatory markers might be explained by the inefficient bioconversion of ALA to EPA and DHA and by the potential competition between ALA and LA for desaturates and elongates (8, 31, 32). Besides, LA has been shown to limit the synthesis of EPA from ALA in humans (8). Thus, the anti-inflammatory effect of ALA might depend on the dietary habits, and a high amount of dietary LA might limit endogenous EPA synthesis, potentially producing a more inflammatory environment.

Examining the change in lipid profile, it is noteworthy that a higher reduction of TC, LDL and TG in the experimental group post-intervention occured. Despite the lack of statistical significance, the authors depict some clinical relevance, which need further studies with a larger sample size to increase discussion and understanding of such “bioactive molecules” that could serve as therapeutic adjuvants of dyslipidemia in dietary management. However, the results obtained are not dissimilar to those of other authors (25, 26), starting from Schwab U.S. et al. (25), who demonstrated the significant reduction of serum levels of TC (p = 0.008) and LDL (p = 0.022) after 12-weeks daily consumption of Camelina sativa oil. Again, Karvonen H.M. et al. (26) reported a significant decrease in serum TC and LDL concentrations after 30 days of oil consumption in all intervention groups (p = 0.001 and p = 0.003 or TC and LDL, respectively), with no significant difference among the investigated oils despite camelina oil has led to a 3–6% LDL-cholesterol lowering effect more than the other two (rapeseed and olive oil) (26).

To the best of our knowledge, this is the first study that aimed to assess long-chain omega-3 fatty acid serum concentrations, inflammatory markers, and lipid profile modification in free-living older subjects through a functional food (Camelina sativa oil-enriched crackers), instead of pure Camelina oil (25, 26). The strength of the current research is the study design: a double-blind, randomized, placebo-controlled trial.

On the other hand, a few study limitations need to be acknowledged, including the small sample size and the impact of the COVID-19. Restrictions affected the progress of this research protocol as well as many others that did not relate to COVID-19 research questions, by discouraging participation in the study and increasing dropout. A per protocol analysis, that excludes patients who deviated from the protocol, was used, but even if this can introduce an attrition bias, dropouts and participating patients had similar characteristics.

Despite the mixed results and considering the burden of inflammageing as a pivotal role in age-related diseases, the authors draw from this study the stimulus to deepen the knowledge of omega-3 fatty acids, specifically from alternative and plant-based sources. In a context where nutrition and sustainability need to be combined to support the prevention of the most common risk factors for Non-Communicable Diseases (NCDs) in an older population destined to increase, Camelina Sativa oil could represent a promising source of ALA.

Evidences have demonstrates that improving intake for certain nutrients may be important in reducing progress of NCDs especially in older population more vulnerable to them, indeed more than 50% older adults have one or more NCDs (33). The role of specific components of foods for NCD risk is acknowledged and nutrition also has a role in disease management.

In clinical practice the possibility to recommend target tailored foods fortified with bioactive molecules, accessible to all is a primary contributor to a long and productive life (33).

Facilitating healthier and longer living through food and nutrition is not just a health agenda, it is crucial for all the agienig sociaty. Multi-stakeholder collaboration will enable to bring scientific innovations and market opportunities to contribute to the prevention of Non-Communicable Diseases, especially in the most vulnerable groups like elderly.

Future research should focus on the discovery and enhancement of plant-derived bioactive metabolites also from waste plant products developing sustainable foods for human well-being. Enriched food is a more attractive alternative to improve health within regular meals, than conventional dietary supplements.

Thus, in an era in which greater awareness of the role of food and nutrition in human health is being achieved a balanced and varied diet, containing foods rich in bioactive compounds, may play a crucial role in the prevention of chronic diseases mediated by inflammatory processes, bearing the promise to bring nutrition to the forefront of clinical medicine.

## Electronic supplementary material

Supplementary material, approximately 59.2 KB.

## Notes

*Ethics:* This study was conducted according to the guidelines laid down in the Declaration of Helsinki and all procedures involving human subjects were approved by the Ethics Committee of the University of Milano-Bicocca (protocol 410LABRA, 09/21/2018). Written informed consent was obtained from all subjects.
