# Supplementary material for: The Effect of Crackers Enriched with Camelina Sativa Oil on Omega-3 Serum Fatty Acid Composition in Older Adults: A Randomized Placebo-Controlled Pilot Trial
Source: J Nutr Health Aging. 2023 Jun 3:1–9. Online ahead of print. doi: 10.1007/s12603-023-1925-x (PMC10238773; doi:10.1007/s12603-023-1925-x)
Supplement: Supplementary file 1 — Supplementary material, approximately 59.2 KB. [file 12603_2023_1925_MOESM1_ESM.docx]

**Table S1**: *Camelina sativa* oil energy and nutrients composition

| ***Camelina sativa* oil** | |
| --- | --- |
| Average nutritional values for 100 g of product | |
| **Energy** | 460 kcal |
| **Saturated fatty acids** | 10.09 g |
| Monounsaturated fatty acids | 30.7 g |
| Polyunsaturated fatty acids | 56.92 g |
| **Linoleic acid** | 16.3 g |
| **α-Linolenic acid** | 36.46 g |
| **Erucic acid** | 3.34 g |
| **Vitamin E** | 0.115 g |

**Table S2**. Concentration of fatty acids in placebo and Camelina groups at T0 and T1.

|  | **Placebo group** | | | | | | | |
| --- | --- | --- | --- | --- | --- | --- | --- | --- |
|  | **T0** | | | | **T1** | | | |
|  | **N** | **mean (SD)** | **median** | **min-max** | **N** | **mean (SD)** | **median** | **min-max** |
| **ALA (µmol/L)** | 31 | 6.8 (3.7) | 5.8 | 2.5 - 18.7 | 24 | 12.8 (6.04) | 11.9 | 3.6 - 23.9 |
| **EPA (µmol/L)** | 31 | 1.1 (0.83) | 0.9 | 0.3 - 4.1 | 24 | 2 (1.55) | 1.4 | 0.5 - 7.3 |
| **DHA (µmol/L)** | 31 | 3 (1.31) | 2.4 | 0.8 - 6.2 | 24 | 6.5 (3.22) | 5.7 | 1.9 - 15.2 |
| **LA (µmol/L)** | 31 | 45.3 (20.38) | 39 | 14.4 - 100.7 | 24 | 72.4 (38.33) | 63.9 | 10.7 - 161 |
| **ARA (µmol/L)** | 31 | 3.9 (1.96) | 3.8 | 1.5 - 9.9 | 24 | 8.5 (3.35) | 7.5 | 4.7 - 17.8 |
|  | **Camelina group** | | | | | | | |
|  | **T0** | | | | **T1** | | | |
|  | **N** | **mean (SD)** | **median** | **min-max** | **N** | **mean (SD)** | **median** | **min-max** |
| **ALA (µmol/L)** | 33 | 6.6 (2.93) | 6.1 | 2.5 - 15.3 | 24 | 18.5 (9.52) | 15.2 | 5.4 - 36.8 |
| **EPA (µmol/L)** | 33 | 1 (0.63) | 0.8 | 0.4 - 3.1 | 26 | 3.4 (4.19) | 2 | 0.6 - 21 |
| **DHA (µmol/L)** | 33 | 3.1 (1.56) | 3 | 1 - 7.6 | 26 | 7 (4.7) | 5.4 | 2.1 – 22 |
| **LA (µmol/L)** | 33 | 46.3 (17.61) | 44.2 | 10.3 - 88.6 | 26 | 83.7 (41.3) | 76.8 | 30.6 - 183.2 |
| **ARA (µmol/L)** | 33 | 4.6 (2.44) | 4.1 | 1.4 - 10.7 | 26 | 8.5 (4.7) | 6.6 | 2.9 - 19.7 |

ALA, α-linolenic acid; EPA, eicosapentaenoic acid; DHA, docosahexaenoic acid; LA, linoleic acid; ARA, arachidonic acid; SD, standard deviation.

Data were reported as mean ± SD, median and minimum – maximum.

**Table S3.** Concentration of inflammatory response markers in placebo and intervention groups at T0 and T1.

|  | **Placebo group** | | | | | | | |
| --- | --- | --- | --- | --- | --- | --- | --- | --- |
|  | **T0** | | | | **T1** | | | |
|  | **N** | **mean (SD)** | **median** | **min-max** | **N** | **mean (SD)** | **median** | **min-max** |
| **IL18 (pg/mL)** | 32 | 273.4 (108.03) | 254.5 | 133.6 - 575.4 | 24 | 245.9  (83.39) | 228.3 | 126 - 439.8 |
| **TNFα**  **(pg/mL)** | 31 | 11.3  (7.78) | 8.5 | 1.8 - 33.3 | 24 | 19.9  (19.8) | 11.3 | 0.01 - 83 |
| **TGFβ1 (pg/mL)** | 32 | 86960.6 (18944.19) | 88875.5 | 49313 - 116608.5 | 23 | 85897 (18696.5) | 87683.8 | 55134.1 - 116355.8 |
| **TGFβ2 (pg/mL)** | 31 | 254.2 (278.97) | 205.3 | 14.4 - 1525.1 | 24 | 165.3 (207.03) | 109 | 0.01 - 933.3 |
| **CRP (pg/mL)** | 31 | 1581444 (1251505) | 1220000 | 144940.7 - 5520000 | 24 | 1272585 (965211) | 1016572 | 240320.2 - 4170000 |
|  | **Camelina group** | | | | | | | |
|  | **T0** | | | | **T1** | | | |
|  | **N** | **mean (SD)** | **median** | **min-max** | **N** | **mean (SD)** | **median** | **min-max** |
| **IL18 (pg/mL)** | 34 | 252.5 (105.8) | 245.2 | 90 - 632.6 | 26 | 254.8 (104.63) | 253.2 | 91.7 - 600.3 |
| **TNFα**  **(pg/mL)** | 31 | 9.7 (7.01) | 7.1 | 2.7 - 29.6 | 26 | 22.2 (16.24) | 21.6 | 0.01 - 71.1 |
| **TGFβ1 (pg/mL)** | 34 | 83671.8 (32311.68) | 76391.4 | 42157.1 - 200976.2 | 26 | 73684.8 (23579) | 65829.4 | 45165.7 - 133800.3 |
| **TGFβ2 (pg/mL)** | 31 | 257.9 (221.71) | 178.2 | 30.9 - 1070.3 | 26 | 161.1 (145.07) | 172.3 | 0.01 - 667.7 |
| **CRP (pg/mL)** | 34 | 1886009 (1854319) | 1035000 | 109128.6 - 7760000 | 24 | 2034691 (1980477) | 1370000 | 151399.4 - 7860000 |

IL18, interleukin – 18; TNFA, tumor necrosis factor-alpha; TGFB1, transforming growth factor-beta 1; TGFB 2, transforming growth factor-beta 2; CRP, C-reactive protein; SD, standard deviation.

Data were reported as mean ± SD, median and minimum – maximum.

**Table S4**. Lipid profile in placebo and intervention groups at T0 and T1.

|  | **Placebo group** | | | | | | | |
| --- | --- | --- | --- | --- | --- | --- | --- | --- |
|  | **T0** | | | | **T1** | | | |
|  | **N** | **mean (SD)** | **median** | **min-max** | **N** | **mean (SD)** | **median** | **min-max** |
| **TC (mg/dL)** | 32 | 201.4 (33.18) | 210.5 | 131 - 247 | 24 | 203.6 (30.74) | 208.5 | 141 - 240 |
| **HDL (mg/dL)** | 32 | 63.8 (13.23) | 62.5 | 34 - 84 | 24 | 63.6 (11.91) | 62.5 | 38 - 87 |
| **LDL (mg/dL)** | 32 | 130.5 (31.68) | 136 | 56 - 178 | 24 | 131.3 (29.11) | 136 | 67 - 178 |
| **TG (mg/dL)** | 32 | 93.4 (29.14) | 91.5 | 51 - 166 | 24 | 91.2 (32.95) | 88 | 47 - 203 |
|  | **Camelina group** | | | | | | | |
|  | **T0** | | | | **T1** | | | |
|  | **N** | **mean (SD)** | **median** | **min-max** | **N** | **mean (SD)** | **median** | **min-max** |
| **TC (mg/dL)** | 34 | 198.8 (41.52) | 202 | 103 - 292 | 26 | 195.2 (37.68) | 207 | 119 - 267 |
| **HDL (mg/dL)** | 34 | 58.6 (14.29) | 57.5 | 32 - 95 | 26 | 56.4 (15.17) | 54.5 | 34 - 96 |
| **LDL (mg/dL)** | 34 | 131.2 (42.71) | 126.5 | 59 - 220 | 26 | 125.4 (38.98) | 118 | 66 - 219 |
| **TG (mg/dL)** | 34 | 117.1 (53.24) | 103 | 35 - 250 | 26 | 118.9 (40.93) | 109.5 | 49 - 198 |

TC, total cholesterol; HDL, high-density lipoproteins cholesterol; LDL, low-density lipoproteins cholesterol; TG, triglycerides; SD, standard deviation.

Data were reported as mean ± SD, median and minimum – maximum.

**Table S5**. Medications

|  | **Total** | **Placebo group** | **Camelina group** |
| --- | --- | --- | --- |
| **Hypertension** | 17 | 6 | 11 |
| **Osteoporosis** | 16 | 10 | 6 |
| **Arthritis** | 10 | 3 | 7 |
| **Hypercholesterolemia** | 19 | 10 | 9 |
| **Type 2 diabetes** | 2 | 1 | 1 |
| **Chronic obstructive pulmonary disease** | 2 | 0 | 2 |
